# NLRexpress—A bundle of machine learning motif predictors—Reveals motif stability underlying plant Nod-like receptors diversity

**DOI:** 10.3389/fpls.2022.975888

**Published:** 2022-09-15

**Authors:** Eliza C. Martin, Laurentiu Spiridon, Aska Goverse, Andrei-José Petrescu

**Affiliations:** ^1^Department of Bioinformatics and Structural Biochemistry, Institute of Biochemistry of the Romanian Academy, Bucharest, Romania; ^2^Laboratory of Nematology, Department of Plant Sciences, Wageningen University, Wageningen, Netherlands

**Keywords:** plant resistance, NLR motifs, NB-ARC predictor, LRRpredictor, NLR correlations

## Abstract

Examination of a collection of over 80,000 Plant Nod-like receptors (NLRs) revealed an overwhelming sequence diversity underlying functional specificity of pathogen detection, signaling and cooperativity. The NLR canonical building blocks—CC/TIR/RPW8, NBS and LRR—contain, however, a number of conserved sequence motifs showing a significant degree of invariance amongst different NLR groups. To identify these motifs we developed NLRexpress—a bundle of 17 machine learning (ML)-based predictors, able to swiftly and precisely detect CC, TIR, NBS, and LRR motifs while minimizing computing time without accuracy losses—aimed as an instrument scalable for screening overall proteomes, transcriptomes or genomes for identifying integral NLRs and discriminating them against incomplete sequences lacking key motifs. These predictors were further used to screen a subset of ∼34,000 regular plant NLR sequences. Motifs were analyzed using unsupervised ML techniques to assess the structural correlations hidden underneath pattern variabilities. Both the NB-ARC switch domain which admittedly is the most conserved region of NLRs and the highly diverse LRR domain with its vastly variable lengths and repeat irregularities—show well-defined relations between motif subclasses, highlighting the importance of structural invariance in shaping NLR sequence diversity. The online NLRexpress webserver can be accessed at https://nlrexpress.biochim.ro.

## Introduction

The extensive repertoire of NOD-like receptors (NLR) in plants, which often encode for so-called resistance (R) proteins, have a pivotal role in the plant’s innate immune system to counteract a wide range of invading pathogens and pests. The co-evolutionary dynamics between pathogen and host led to a vast expansion of NLR interaction networks and operational mechanisms performed by this class of proteins sharing a more or less similar structural organization (Jones et al., 2016; Schreiber et al., 2016; Shao et al., 2016; Sukarta et al., 2016; Baggs et al., 2017; Wu et al., 2017; Cesari, 2018; Maruta et al., 2022). Canonical NLRs consist of a variable N-terminal domain, a central nucleotide-binding NBS domain and a leucine-rich repeat LRR domain (Sukarta et al., 2016). However, extensions up- and down-stream of this core region are often present and variable domains are even integrated between these core subunits to provide functional specificity to the protein (van der Hoorn and Kamoun, 2008; Cesari et al., 2014; Wu et al., 2015; Khan et al., 2016; Kroj et al., 2016; Andolfo et al., 2019).

Sequence variability of these proteins in the N-ter region led to a broadly accepted classification into several NLR groups. The largest of these are the TNL and CNL classes displaying toward their N-ter end either a toll/interleukin-1 receptor (TIR) or a coiled-coil (CC) domain, respectively. TIR domains are well-conserved structures spanning across the entire tree of life which display the so-called Rossman fold also known as the “ADP-binding βαβ fold” due to the ability to bind the ADP region of dinucleotides (Hanukoglu, 2015). TIR domains are common to several immune system proteins in higher eukaryote organisms. While highly conserved in plant TNLs, the plant TIR group diverges significantly from metazoan TIRs with distinctive, thoroughly analyzed features (Toshchakov and Neuwald, 2020) including specific additions and ablations which make them prone to a precise identification. This high TIR conservation in plant TNLs is in startling contrast to the CNL diversity in which the N-ter CC domains exhibit a very high variability. Most CCs display 4 or 5 predicted helical segments of which the 3rd is the most conserved, embedding the so-called EDVID motif which was proved to have critical functions in several systems (Rairdan et al., 2008; Maekawa et al., 2011; Hao et al., 2013; Casey et al., 2016; Slootweg et al., 2018; Wróblewski et al., 2018) and/or to be involved in CC-LRR interaction as shown by ZAR1 cryo-EM structures (Wang et al., 2019a,b). Among other, less frequently reported extensions in the N-ter region of NLRs were SD, kinase, α/β hydrolase, WRKY (Sarris et al., 2015; De Oliveira et al., 2016; Kroj et al., 2016; Andolfo et al., 2019).

In all NLRs, the most conserved region is the central NB-ARC/NBS domain which acts as an “on/off switch” that changes its configuration upon activation by a matching pathogen effector (Sukarta et al., 2016; Burdett et al., 2019; Wang et al., 2019a; Ma et al., 2020). In plants this domain consists of three subdomains: the Nucleotide Binding Domain (NBD), the Helical Domain, HD1, also known as ARC1 and the Winged Helix Domain, WHD, also termed ARC2. In the inactive state, the three subdomains collectively bind an ADP molecule. During activation, ADP is released and replaced by an ATP. This leads to a drastic change of configuration in which the ARC2 rotates ∼180° with respect to NBD/ARC1. The nucleotide-binding pocket is formed by residues located in seven highly conserved regions spread all over the three NBD, ARC1 and ARC2 subdomains, referred to herein as: VG, P-loop, Walker B—in NBD; RNBS-B, RNBS-C—in ARC1 and GLPL, MHD—in ARC2 (Tameling et al., 2002; Van Ooijen et al., 2008; Qi and Innes, 2013; Wróblewski et al., 2018; Johanndrees et al., 2021; Yu et al., 2021). Along with these seven “pocket” stretches two other regions which are instrumental in providing subdomain stability show a rather high invariance: the RNBS-A in NBD and RNBS-D in ARC2 (Wang et al., 2019b; Ma et al., 2020; Martin et al., 2020b).

Finally, the LRR domain has a solenoidal, horseshoe-like 3D architecture and consists of recurrent stretches of 15–35 amino acids length that start each with a so-called LxxLxL motif, where L is a hydrophobic amino acid, dominantly leucine, and X can be any residue (Kajava, 1998; Kobe and Kajava, 2001). Repeats are held together via a H-bond network formed among the LxxLxL stretches located on the ventral side of the horseshoe. The LRR domain is present not only in NLRs, but in many other immune system proteins, both in plants and metazoans. For instance, in plant Receptor-Like Kinases/Proteins (RLK/RLP) and metazoan TLRs the LRR domain is localized on the extracellular side of the receptor and is responsible for the direct detection of pathogen-associated molecules (Sun et al., 2013; Rajaraman et al., 2016; Eckardt, 2017; Dievart et al., 2020). Some of these RLKs require also the association with other membrane-bound receptors through their own LRR domains such as FLS2-BAK1 (Sun et al., 2013). In cytosolic NLRs, the LRR domains were shown to display various intra- and inter-molecular interacting functions. For example, the cryo-EM structures of ZAR1 CNL and RPP1 TNL (Wang et al., 2019a,b; Ma et al., 2020) indicate that the N-ter region of the LRR interacts with NBS. On the other hand, while in ZAR1 the downstream C-ter region of the LRR binds to the RKS1 kinase which is an adaptor for the recognition of the effector, in other NLRs such as RPP1, Rx or Gpa2, the LRR domain recognizes directly the effector or the pathogen molecule (Slootweg et al., 2017; Ma et al., 2020).

The working environment imposes interesting constraints on the local properties of LRR sequences. Whereas the extracellular LRR domains of RLK/RLP/TLRs display repeats of regular ∼24 aa lengths and almost invariant 3D shape, in cytosolic NLRs LRR repeats are very irregular while the LxxLxL motifs are highly variable and ambiguous, with many local alternative “satellites” (Kobe and Kajava, 2001; Kajava and Kobe, 2002; Sela et al., 2012, 2014; Baudin et al., 2019; Wang et al., 2019b). This imposes a high level of uncertainty in repeat delineation which makes the investigation of structure-function relations problematic and inflicts on model-driven analysis of LRR fold stability, interdomain and protein-protein interactions. Some more accurate machine learning-based tools were recently developed to address this problem (Bej et al., 2014; Martin et al., 2020a; Liu et al., 2022) but these are computationally costly. For instance, the LRRpredictor (Martin et al., 2020a), which was specifically designed to address motif irregularities, relies on the consensus of 8 classifiers of which 4 are based on variability profiles only, built starting from global databases, and 4 rely in addition on secondary structure predictions. Such complex methods clearly require high computational resources which makes them less usable in genome-wide or large proteome datasets scanning.

In this context lighter but similarly precise tools—able to screen overall transcriptomes and proteomes are needed in order to discriminate between complete transcripts and incomplete sequences lacking key functional motifs. To this end we introduce here NLRexpress—a bundle of 17 ML-based predictors designed to identify CC-/TIR-/NBS- and LRR-specific motifs. The main focus in developing NLRexpress was to create an instrument scalable for screening large sets of plant NLRs based on simple but effective NN models able to reduce the computing time with minimal accuracy losses. Motifs identified with NLRexpress in a large NLR atlas (NLRscape, 2022) were then subjected to unsupervised classification in order to identify correlations hidden underneath motif variabilities in individual domains and the overall NLR structure.

The online NLRexpress webserver can be accessed at https://nlrexpress.biochim.ro and the standalone version can be downloaded from https://github.com/eliza-m/NLRexpress.git.

## Materials and methods

### Training sets and preprocessing

In generating the training sets for NLR motifs we used data found in the NLRscape database which organizes information on over 80,000 plant NLR proteins and fragments (NLRscape, 2022). From the overall set, sequences that contain a single, complete canonical NBS domain with a proper NBD-ARC1-ARC2 subdomain layout were clustered at 90% identity and 90% coverage resulting in a subset of ∼34,000 NLR. This contains around 41% CNLs (CC-NBS-LRR), 15% TNLs (TIR-NBS-LRR), and 1.2% RNLs (RPW8-NBS-LRR) while the remaining 43% sequences are either NLs (NBS-LRR) or display incomplete or a non-canonical domain layout.

Individual CC, TIR, NBS, and LRR domain regions were then extracted from the full protein sequence and further clustered at a minimum of 70% domain coverage and various sequence identity thresholds using MMseqs2 (Steinegger and Söding, 2017).

For the CC domain, a 30% identity threshold was imposed resulting in 685 representatives. This set was further manually curated to eliminate incomplete CC entries with large deletions of 15–20 aa in helical predicted regions and retain only those entries having CC lengths of over 120 aa including the linker to NBS. By this, a final clean set of 475 sequences was retained for EDVID CC motif training.

A similar approach was used in generating the TIR motifs training data. By contrast to the CC domains which are highly variable, TIRs are significantly more conserved—therefore a 60% identity cutoff was enough to obtain a set of 881 representatives. After the manual inspection and elimination of incomplete motifs, 490 sequences were retained for training the TIR module.

As the NBS region displays not less than nine highly conserved potential motifs (VG; P-loop; RNBS –A, –B, –C, and –D; Walker B, GLPL, and MHD) spread over the three subdomains of NBS, only sequences displaying the complete NBD-ARC1-ARC2 layout were selected. By using a cutoff of 50% sequence identity at 70% coverage, a set of 1,910 representatives was obtained, which was further reduced to 861 by the elimination of incomplete N-/C- termini or missing motifs sequences.

Finally, for LRR motif detection, a curated set of 117 LRR domains containing ∼2,000 core LRR motifs was selected for the overall training set resulting from the following workflow. In a first step, only sequences containing both complete NBS and LRR domains were retained. The LRR domain regions of these sequences were clustered at 50% identity and clusters were ranked according to their dimension. From these, only the top 117 clusters were retained, containing each over 40 sequences while the rest were treated as sparse LRR outliers. Sequences within the top 117 largest clusters were further subjected to LRR motif delineation with LRRpredictor (Martin et al., 2020a) and in-depth sequence profiling and analysis for representative selection. While the level of intra-cluster identity ensures a robust structural homology, the sequence diversity induces local variations of profiles such as the secondary structure state or the LRR motif probability—these were in turn used as selection criteria in choosing the optimal cluster representative.

### Feature selection

In most state-of-the-art sequence-based predictors, variability profiles are computed based on remote homologs retrieved from very large databases such as Uniprot20 or Uniclust30/50. However building such HMM/PSSM profiles starting from large databases comes with a significant computational cost, especially when the protein query contains repetitive patterns such as LRR motifs—due to the vast increase in multiple alignment possibilities of such repetitive stretches found in the hits of a given queried sequence. As this is the time-limiting step in generating the NLR profiles, instead of using the Uniprot/Uniclust datasets, we explored the possibility of compiling downscaled custom-generated focused sets in achieving the best tradeoff between the computational cost and the HMM profile relevance. To this end, in building profiles, a set of 2,361 sequences was generated consisting of a collection of 1,361 plant NLRs of at least 500 amino acids filtered at 20% identity and an extra group of 1,000 LRR domains larger than 200 aa filtered at the same identity level, but derived from different protein types and origins—added in order to allow the predictor to detect swiftly in a given genome all kinds of LRRs, not only those NLR specific. This focused dataset was further used by all the 11 predictors included in NLRexpress to generate the training features which consist of match emissions probabilities inferred using the JackHMMER suite (Johnson et al., 2010; Potter et al., 2018) with a 1e-5 expect value cutoff. The first and second iterations of HMM profiles were investigated as features and using them in conjunction proved to yield the best results.

### Network architecture—Training and performance evaluation

Supervised classifiers were trained for each of the 17 individual motif types—specific to the CC (extended EDVID), TIR (βA, αA, βC, αC, βD-αD1, αD3), NBS (VG; P-loop; RNBS –A, –B, –C, and –D; Walker B, GLPL, MHD) and LRR (LxxLxL) domains. For each residue in the input sequence, the individual predictors compute the probability to be the starting point of a motif by using as features the negative natural log of HMM emission probabilities to each 20 possible amino acids computed in the first and second iteration over a sliding window of 5 positions upstream and downstream motif borders adding up to a total number of 20 × 2 × (5 + motif length + 5) input features.

For training the individual predictors, the CC, TIR, NBS, and LRR motif datasets were split into 5 equal groups. The first 4 groups were used for parameter optimization using a fourfold cross-validation scheme, while the fifth group was left aside and used as the final test set. In order to tradeoff between class imbalances, non-linear separable problems on small datasets and computational costs, a multilayer perceptron network (MLP) architecture, known to effectively mitigate such issues, was chosen as the supervised algorithm. The hyperparameter tuning stage was conducted using a fourfold cross-validation scheme in which the number of hidden layers, neurons per layer, the solver and regularization parameters were subjected to optimization. A network with three hidden layers of 250, 125, and 100 neurons, respectively, was found to optimally suit all the 17 classifiers, while the Adam stochastic gradient-based optimizer (Kingma and Ba, 2015) with a constant learning rate of 0.001, ReLu activation function and L2 penalty regularization with customized alpha parameters for each predictor were selected as network parameters. The out-of-sample performance was evaluated on the test set using the precision, recall, specificity, F1 and G scores metrics. For training and evaluating the predictors, the scikit-learn library v24.2 (Pedregosa et al., 2011) was used.

### Nod-like receptor motifs analysis

NLRexpress was then used to predict NLR motifs on the overall set of ∼34,000 NLRs trimmed at 90% identity. As the overall database contains not only regular NLRs but also all sorts of non-canonical sequences with missing, duplicated or shuffled NLR domains, selections were performed to derive subsets fulfilling specific criteria in order to carry out more targeted correlation analysis.

For instance, in order to identify correlations within NBS motifs a subset of only ∼20,000 sequences was selected containing at the same time all the nine motifs—only once, in the right order and with a probability estimate higher than 80%. Extended regions conveying each motif and 5 flanking positions on both sides were then extracted and concatenated in the right order. These excised NBS chimeras were subjected to sequence-based clustering using MMseq2 (Steinegger and Söding, 2017) at various identity thresholds using the connected component clustering method as implemented in the MMseq2 suite. Correlation analysis was performed using the CMAT method (Jeong and Kim, 2012) as implemented in VisualCMAT (Suplatov et al., 2018) and MISTIC (Simonetti et al., 2013). Henikoff weights and profile-based pseudocounts were used for join probability adjustments. A minimal Z-score of 3.5 was set as cutoff for both cumulative and proximity mutual information metrics.

For LRR motif distribution analysis domains were selected only if: **(a)** they contain upstream a proper NBS domain with all 9 motifs unambiguously identified in the right order; **(b)** they display the LRR domain downstream NBS with a linker less than 50aa; **(c)** they contain at least 9 LRR repeats of expected regular lengths: 15–50 aa. This last constraint was imposed to avoid ambiguous motif assignments. An exception was made though in cases where the difference in probability among local motif “satellites” found in less than 15 aa surpasses 20%—a situation in which the motif with the highest probability was selected. With these cleaning constraints, a subset of ∼6,850 regular NLR sequences were selected containing a total number of ∼61,700 LRR motifs.

The LRR motifs were further clustered based on the physico-chemical properties of amino acids in the LxxLxL region. For each of the six positions the hydropathy, charge and volume were used as descriptors summing up to an 18-dimensional clustering embedding. Given its ability to deal with uneven cluster sizes, densities and non-flat geometries the OPTICS algorithm (Ankerst et al., 1999) was selected from several unsupervised clustering methods tested, and ran using its scikit-learn library implementation along with the Xi algorithm as the extraction method and the Minkowski norm 2 metric (Pedregosa et al., 2011).

For the analysis of the N-ter NLR region, TNL/CNL, a sequence-based clustering approach was used similarly to the NBS domain analysis. In the case of TNLs, a subset of 3,774 sequences was gathered to contain a TIR domain with all 6 TIR motifs unambiguously identified in the right order and a downstream proper NBS domain with all 9 NBS motifs. The extracted motif regions with 5 flanking positions on both sides were concatenated and subjected to sequence-based clustering using MMseq2 (Steinegger and Söding, 2017). For CNLs, in addition to the above-mentioned criteria imposed to identify regular NLR proteins, a supplementary condition was used regarding the presence of a single integral CC domain upstream of the NBS. Given that all the potential CNL interdomain interactions were shown to take place within the first five LRR repeats (Qi et al., 2012; Slootweg et al., 2013, 2017; Wang et al., 2019b) CNLs displaying at least five proper repeats, not nine as in LRR domain analysis, were selected resulting in a clean 6,000 CNL subset which was further subjected to a workflow similar to the one described above. As in the NBS analysis—regions covering all the 15 motifs within the CC, NBS and the first five N-ter LRR, each with 5 flanking positions on both sides were extracted and concatenated in the right order to be subjected to sequence-based clustering using MMseq2 (Steinegger and Söding, 2017) with various identity thresholds. Logomaker (Tareen and Kinney, 2020) was used to generate sequence variability plots, while figures illustrating 3D protein structures were edited using (PyMOL, 2018; The PyMOL Molecular Graphics System, Version 2.2.3 Schrödinger, LLC).

## Results and discussion

### NLRexpress structure, development, and performance analysis

**FIGURE 1:**
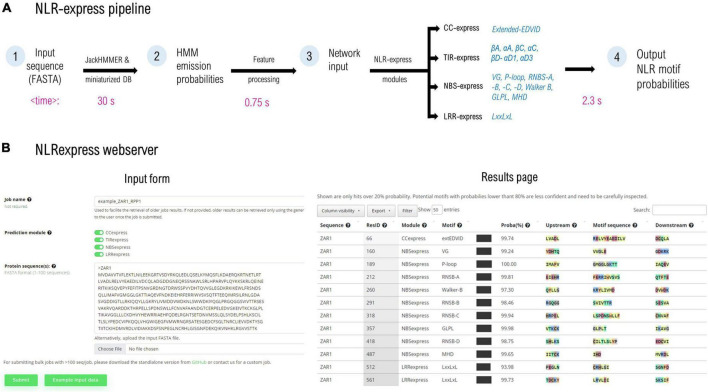
**(A)** NLRexpress pipeline diagram with typical computation times shown below for a queried NLR sequence of average length. **(B)** Screen capture of the NLRexpress webserver: input form and the results page containing the prediction output. Average times may vary depending on hardware and sequence properties.

NLRexpress consists of a collection of 17 NN-based predictors trained to identify individual plant NLR domain-specific motifs and it is organized as four prediction modules: CC-, TIR-, NBS-, and LRRexpress that can be also used independently. The CC- and LRRexpress modules contain a single motif predictor for the extended EDVID and LxxLxL pattern respectively, while TIRexpress comprises a bundle of 6 motif predictors for the following most preserved elements: βA, αA, βC, αC, βD-αD1, αD3, and NBSexpress contains a bundle of 9 individual motifs for the following conserved regions: P-loop; RNBS –A, –B, –C, and –D; Walker B, GLPL, and MHD (Figure 1A).

The workflow of NLRexpress is shown in Figure 1. As input, the predictor accepts amino acid sequences provided by the user in FASTA format. In a first step which is also the most time-consuming, NLRexpress generates the numerical features required by the NN models. Next, the NN-model input is processed and fed to the prediction modules. Users can select either a single CC/TIR/NBS/LRR module or all four together. The output consists of a probability estimate for each residue of the sequence to be the starting position of the given motif (Figure 1B).

For generating the features, HMM profiles are usually learnt from a set of homologs queried over a protein sequence database. Today state of the art predictors generally rely in this step on global protein DBs such as Uniprot20 (∼30 GB; ∼8.3 million sequences), and Uniclust30 (∼9 GB; ∼10 million sequences), the scanning of which results in high computational costs. In order to reduce the execution time of this stage, NLRexpress alternatively relies on a downscaled, manually curated dataset. This ensures an optimal tradeoff between performance and speed. For instance, using the downscaled dataset reduces eightfold the time required to build the HMM profile for ZAR1 using Uniclust30, at the same accuracy. However, even when starting from such small datasets of only ∼2,400 seq, the longest duration of feature generation is 15-fold higher than the rest of the workflow including predictions—which take on average less than 3.5 s per protein (Figure 1A).

As described under methods, curated sets of CC, TIR, NBS, and LRR domains were used to train and test the 17 individual NN models. The four domain sets were divided into five subsets of which the first 4 were used in a fourfold cross-validation scheme for parameter tuning while the fifth was left aside for a final out-of-sample predictor performance testing. The workflow results in four predictor modules for the CC, TIR, NBS, and LRR motifs, respectively.

**FIGURE 2:**
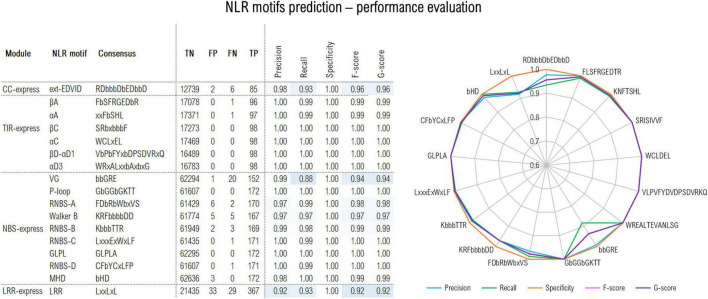
Out-of-sample performance of the NLRexpress predictors on the test set (TP, true positive; TN, true negative; FN, false negative; TP, true positive). Cross-validation out- and in-sample performance results are shown in Supplementary Table 1.

The CC predictor focuses on the EDVID motif found in many CC domain classes. The initial training trials were performed using the minimum EDVID motif span and proved less effective. However, by using an extended version of the motif spanning over 12 positions covering the upstream region involved in the CC-LRR interfacing in the ZAR1 cryoEM structure (Wang et al., 2019a,b)—with *RDbbbDbEDbbD* as consensus (b-hydrophobic residues)—significantly improved the F1 score to a proper 96% value (Figure 2).

The TIR module identifies six highly conserved plant TNLs regions (Toshchakov and Neuwald, 2020): βA, αA, βC, αC, βD-αD1, αD3 (where α and β refer to helical and beta-strand elements and A-D correspond to structural modules of the TIR domain). Given their high conservation, predictors attain almost perfect performance with F/G scores above 99% (Figure 2).

The NBS predictor targets 9 motifs: *P-loop; RNBS –A, –B, –C, and –D; Walker B, GLPL*, and *MHD*. The highest scores were reached by the most conserved NBS motifs with precision, recall and specificity and F/G scores surpassing 96% (Figure 2). A somehow lower sensitivity/recall of only 88% was shown by the VG motif which is expected given its higher variability. Detailed stats on the cross-validation *out-of-/in-sample* performance can be examined from Supplementary Table 1.

Finally, LRRexpress focuses on the detection of *LxLxxL* motifs *in LRR domains in general*. This yields a total F-score of 92% on the test set, with a balanced precision to recall ratio. As an additional provision ZAR1, ROQ1 and RPP1 clusters were set aside from the training data and used only in the out-of-sample predictor performance testing as these are so far the only plant CNLs/TNLs with known full-protein 3D structure. LRRexpress is able to accurately identify 12/13 repeats in the case of ZAR1, while 19/20 and 21/21 in the case of RPP1 and ROQ1, respectively, which is consistent with the ∼90% sensitivity estimated on the test set.

While the out-of-sample results assessed above involve only plant NLRs, the extrapolation ability and performance of LRRexpress were next investigated on other protein classes containing LRR domains.

Using a set of 178 structures from PDB containing LRR domains at 90% sequence identity, consisting of ∼2,000 manually curated and delineated LRR repeats from Martin et al. (2020a), LRRexpress reaches an F1 score of 92% when considering only the core LRR motifs and 88% when the border, more diverse N-ter and C-ter motifs are included (Supplementary Figure 1A). Compared to the plant NLR set, the precision/recall ratio is much more tilted toward higher precision rates (98%) rather than sensitivity (88% on core motifs and 80% when border motifs are included).

In order to further evaluate the LRRexpress performance a thorough comparison with a former more laborious method implemented in LRRpredictor (Martin et al., 2020a) was undertaken. For this LRRexpress was run on the same set used previously for testing the LRRpredictor out-of-sample performance. While this yields a somehow lower F-score when compared to LRRpredictor, ∼85% vs. 90%, it displays on the other hand a higher precision, 99% vs. 92%. Results indicate that LRRexpress shows an overall 88–99% agreement rate with LRRpredictor in pinpointing the same LRR motifs on various LRR-containing protein classes: cytosolic plant resistance proteins (CNL and TNL), extracellular plant receptors with or without kinase domains (RLK/RLP) and their vertebrate corresponding classes—cytosolic NLRs and extracellular TLRs (Supplementary Figure 1C). However, the cross-diagonal cases where the two predictors disagree have a large proportion of either atypical motif patterns or result from ambiguities related to motif selection in regions showing multiple local alternatives.

To assess its discriminative capacity, LRRexpress was also tested using as inputs other solenoidal architectures containing high levels of LxxLxL patterns that might result in false-positive predictions. On five benchmark sets each comprising 50 sequences from ankyrin, armadillo, trimeric, and pectate lyases classes from Martin et al. (2020a), LRRexpress is able to correctly classify the LxxLxL patterns as non-LRR motifs, with almost no false positives in 1,000–2,700 LxxLxL patterns per set (Supplementary Figure 1B).

NLRexpress was further subjected to speed benchmarks on two sets of sequences: (–1) the entire *A. thaliana* proteome consisting of ∼137.000 sequences from UniprotKB; and (–2) A random set of 1,000 plant NLRs retrieved from the overall set. These were subjected to the NLRexpress pipeline for identifying NBS and LRR motifs, on an Intel(R) Core(TM) i9-9900K CPU machine running in three parallel jobs of 4 CPU threads each. The whole Arabidopsis proteome was screened in 31.5 h yielding 930 sequences with complete nine-motif NBS and 4,461 sequences with LRR domains (at least 5 repeats). Given that variability profiles are generated in NLRexpress starting from a reduced and targeted set of clean NLRs supplemented by LRRs from diverse sources—during a genome/proteome/transcriptome scanning the program swiftly discards, in less than 2 s/non-NLR-sequence the “non-NLR/LRR” regions spending most of the time in building profiles of the regions embedding the NLR-specific domains—arr. 32 s/NLR-sequence on average over the 1,000 NLR set, which was scanned entirely in ∼9 h by a 4 CPU thread job.

These results indicate that the LRR module of NLRexpress and LRRpredictor complement each other—given that LRRpredictor is structure-oriented while LRRexpress is sequence oriented, based on a “clean” set of examples—and in conjunction are able to generate an accurate description of LRR domains for structural modeling. On the other hand, this comes with a clear speed advantage for LRRexpress which in addition is able to scan entire genomes/proteomes/transcriptomes for identifying all LRR domains in a reasonable time.

### Motif analysis

NLRexpress was then next used to analyze the overall set of ∼34,000 NLR and NLR fragments at 90% identity in order to identify the main patterns and correlations shaping up in NLR proteins at both individual-domain and global NLR levels.

As the 17 motifs are the main invariants of an NLR sequence these were subjected to clustering, based both on amino acid sequence with Blosum-derived metrics and on physio-chemical properties such as hydropathy, side-chain charge and size.

While distant in sequence, some of these motifs are or might be brought close in space by folding or during the NLR functioning cycle. For instance, seven of the NBS motifs are brought together to form the ADP/ATP binding site of the domain, moreover, the CC/TIR, NBS, and LRR might be permanently or transiently in contact with each other in given functional states of an NLR. Therefore the motif analysis was extended to investigate potential correlations between groups of motifs according to the functional context.

#### The coiled-coil extended EDVID motif

Within the overall set of ∼34,000 NLR and fragment sequences, the CCexpress predictor was able to identify with over 80% probability ∼22,800 extended-EDVID motifs in 20,000 sequences. This excess is due to the fact that many sequences in our database have a more complex domain layout than that of a canonical CC-NBS-LRR layout, with repeated, multiple and/or shuffled arrangements of CC, NBS, LRR, TIR. By focusing now exclusively on the canonical CNL (X-CC-NBS-LRR-X) subset consisting of ∼13,150 sequences 97% display such extended-EDVID motifs (12,815).

#### The six toll/interleukin-1 receptor motifs

**FIGURE 3:**
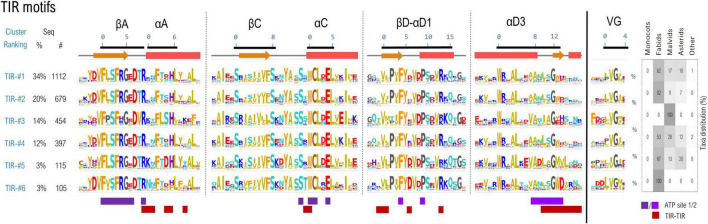
Sequence clustering of the TIR domains based on the confined motif regions. Motif variability is expressed as relative entropy displayed as letter height, while the taxonomic distributions of each cluster is depicted in the right panel. The ATP contacts within 5Å are mapped below the motif consensus (light/dark purple—ATP binding sites 1 and 2 PDB: 7crc). Amino acids are colored according to their properties as follows: yellow, hydrophobic; orange, aromatic; brown, cysteine; cyan, polar; red, acidic; blue, basic; gray, proline, and glycine.

A set of 3,774 TNL sequences which simultaneously contain all 6 TIR and 9 NBS motifs was gathered, and the excised motif sequences with a margin frame of five residues were concatenated and subjected to clustering as described in methods. Using a threshold of 55% identity, around 75% of the TNL sequences cluster in top six largest clusters (Figure 3).

The cryo-EM structures of RPP1 and ROQ TNLs (Ma et al., 2020; Martin et al., 2020b) posit the TIR domains in an asymmetrical tetrameric configuration, binding two ATP molecules between TIRs type 1 and 2 conformation. The nucleotide-binding site 1 consists of βA strand and αC helix and are highly conserved among all TIR classes—particularly the catalytic glutamate and neighboring cysteine in positions 1&4 of αC motif residue experimentally proved essential for NAD* hydrolysis and/or cNMP synthetase activities (Ma et al., 2020; Lapin et al., 2022; Yu et al., 2022). The second ATP binding site is located on the neighboring TIR and consists in (i) the βD-αD1 beta-loop-helix, particularly with conserved Phe and Pro in positions 4 and 9 respectively and (ii) the helical element αD3 and the downstream loop (positions 8–14), more variable across various TNL classes. Besides binding ATP, the regions αA, αC, βD-αD1, and αD3 also participate in TIR-TIR interfacing in the tetrameric structure, and display a higher variability between the identified clusters, suggesting a coevolution process.

#### The nine NBS motifs

**FIGURE 4:**
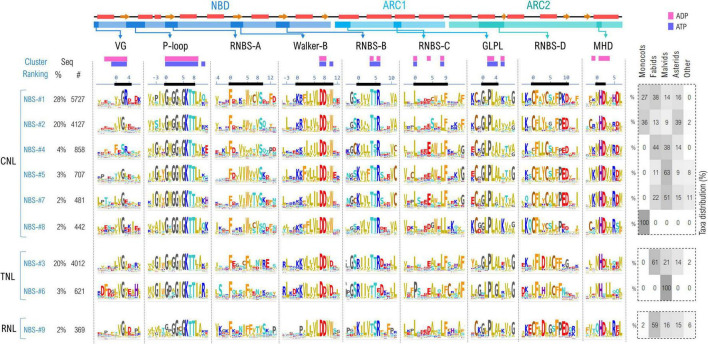
Sequence clustering of NBS domains based on the confined 9 NBS motifs. Motif variability is expressed as relative entropy displayed as letter height—the larger the more conserved. Top clusters are grouped according to the preponderant NLR class: CNL, TNL, or RNL. The taxonomic distributions of each cluster is depicted in the right panel. The ADP/ATP contacts within 5Å are mapped above the motif consensus (magenta—the inactive ADP-bound ZAR1 PDB: 6j5w; purple—the activated ATP-binding ZAR1 PDB: 6j5t). Amino acids are colored as described in Figure 3 caption.

The above-mentioned sequence complexity also inflicts upon the NBS subdomain integrity and organization—as some of the sequences show missing or duplicated regions containing one or multiple NBS motifs. Therefore, the overall set was cleaned to retain only those sequences that contain simultaneously all of the 9 NBS motifs, predicted by NBSexpress, in the right order, with a probability higher than 80%. This reduces the initial set to around 20,000 sequences, with a similar rejection rate of ∼60% as that seen when curating the NBS training set. The 9 motifs were then simultaneously clustered based on their amino acid sequence at different identity thresholds. At 55% identity, 85% of the NBS motifs are conveyed into the top 9 clusters, the largest one comprising ∼28% of all sequences (Figure 4). Interestingly this clustering method, based exclusively on the sequence information confined only within the 9 NBS motifs also adequately separates between NLR types suggesting that differences in the molecular environment in which NBS is confined are a driving force in diversification.

As expected, the most invariant motifs are those containing amino acids forming the ADP/ATP binding sites—especially the P-loop region, Walker-B β-strand, RNBS-B, GLPL and MHD—which is consistent for instance with mutagenesis experiments results on NRC1, SW5, I-2, Mi-1, etc., indicating that many critical single amino acid substitutions are located in the NBS highly conserved motifs (Tameling et al., 2006; Van Ooijen et al., 2008; Sueldo et al., 2015; de Oliveira et al., 2018; Wang et al., 2020). By contrast, the VG, RNBS-A, –C, –D motifs are more diverse, with cluster-specific characteristics.

Of these, the most diverse is the VG motif (*bbGRE*), located right at the border between the CC and NBS domains. In all clusters, the consensus extends with two more hydrophobic positions, denoted by *b*, upstream VG: *bbVG*. The cryo-EM structure of ZAR1 shows that these *bb*V are in contact with the nucleotide in both resting and activated states. The next two positions downstream of the conserved glycine (positions 3 and 4 in Figure 4) are cluster-specific: *VGRE/D* in the largest CNL cluster (NBS #1), while *VGIE/D* (NBS #2,#5), *VGEx* (NBS-#7) in the other CNL clusters. The most atypical is cluster NBS-#4 which displays a significantly divergent motif *FESR*, in which the glycine is replaced by a serine. Oppositely, in the case of TNLs and RNLs the motif consensus is robust—*VGIE/D*—with a hydrophobic and acidic residue downstream of the VG (positions 3, 4; Figure 4).

Next in sequence is the P-loop motif (also termed Walker-A with consensus *GbGGbGKTT*) which is highly invariant across the identified clusters, with modest variations at given positions. Position —1 is either populated by hydrophobic aliphatic (mostly valine) or aromatic residues (tyrosine—in several CNL groups or tryptophan in TNLs). Position 1, which is majorly composed of aliphatic hydrophobic residues, usually methionine, is frequently replaced with proline in a small cluster of TNLs (NBS-#6) found only in malvids clade. The most notable differences are spotted in the RNLs group, where the P-loop motif is less stringent, especially in positions 0–2 (Figure 4).

The highly hydrophobic motif RNBS-A is located in the central, second beta-strand of the NBD and has a structural role in stabilizing strands 1 and 3 which both contribute to the formation of the nucleotide binding site. While quite variable across clusters, a strong preference shapes up for Phe in position 0 of all clusters, with the CNLs Trp in position 5 replaced by Phe in TNLs and RNLs (Figure 4).

Walker-B is located in the next beta-strand. As being involved in nucleotide binding, this motif—*KRFbbbbDDbW*—is highly invariant. Here, letter *b* denotes hydrophobic residues which are mostly aliphatic with the exception of the last one, which in CNLs is frequently a tryptophan (Figure 4).

As also being involved in ligand binding, the RNBS-B motif—K*bbb*TTR—is also highly conserved regardless of the NLR type, with variability occurring only in the flanking regions. Notably, in the small cluster of malvid TNLs (NBS-#6) the arginine in position 6 is less conserved, which is interesting as this position is expected to actively participate in binding the nucleotide (Figure 4).

Within the RNBS-C motif (consensus L*xxx*ExW*x*LF), only the leucines from positions 0 and 8 are highly conserved as they are part of the binding site, while the surrounding positions of the motif are subjected to increased variability. This can be explained by the location of the motif, which covers the NBD-ARC1 linker and the beginning of the first helical segment of ARC1 subdomain (Figure 4).

The GLPL motif is very well conserved across NLR classes, as being a key region in forming the binding site. Notable particularities that can be mentioned are the increased preference for cysteine at position –2 in CNLs and RNLs compared to TNLs in which this cysteine is less conserved.

The RNBS-D motif—CFbYCxLFP—is not involved in binding the ligand but in NBS-LRR interface and shows the highest divergences across the NLR classes. Common to all three NLR classes seems only the high occurrence of Phe in positions 1 and 7. Interestingly positions 0 and 4 appear to show class-specific preference—with two Cys in CNLs; Cys and Leu, respectively, in RNLs; and Leu and Ile in TNLs. In addition the triad Pro-Glu-Asp shapes up in CNLs and RNLs but not in TNLs (Figure 4).

Finally, the MHD motif is well conserved across all NLR classes, whereas variations occur in regions next to the motif. Interestingly, only the NBS-#6 small cluster on TNLs does not have the aspartic acid conserved in position 2, while in RNLs, the methionine in position 0 is often substituted by glutamine (Figure 4).

**FIGURE 5:**
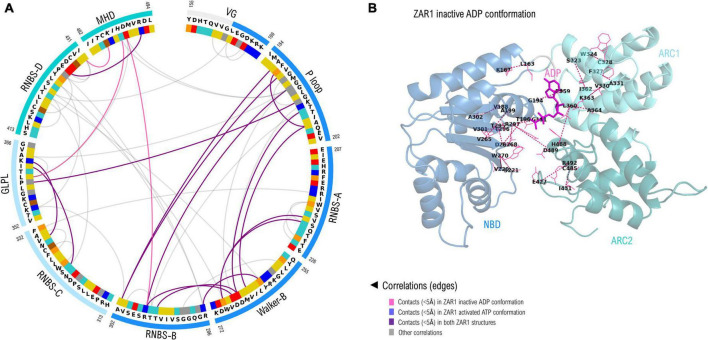
**(A)** Correlated amino acid pairs within the nine NBS motifs. Correlations endorsed by 3D space proximity (<5Å) in ZAR1 inactive ADP cryo-EM structure (6j5w) are shown in violet, while contacts found also in pentameric ATP structure (6j5t) are shown in purple. Correlations not supported by 3D proximity are depicted in gray and were computed using VisualCMAT (Suplatov et al., 2018). Amino acid color code is detailed in Figure 2 caption. **(B)** Correlated interaction pairs mapped onto the cryo-EM inactive ZAR1 structure (PDB: 6j5w). Amino acid pairs are depicted with dotted lines. Domain color code is as in **(A)**: NBD, blue; ARC1, light blue; ARC2, teal.

As seven out of nine NBS motifs are brought close in space in forming the nucleotide binding sites, a correlation analysis was performed in order to identify co-evolving residues. Only the close-in-space correlated pairs were further analyzed and shown in Figure 5, which are numbered according to their positions in ZAR1. As can be seen from Figure 5 most of these close-in-space, correlated positions are found in the NBD subdomain. This can be explained by its large size but also by the beta-sheet architecture that brings close in space residues found far apart in sequence in a more intricate way. Most significant inter-subdomain correlations shape up between the P-loop (NBD) and GLPL (ARC1); GLPL (ARC1) and MHD (ARC2) and between RNBS-B (NBD) and MHD (ARC2). Notably, the correlation identified between residue 297 in RNBS-B “*bbbTTR*” and the ARC2-MHD-489, forms in the ZAR1 structure a saline bridge (R297-D489).

#### The leucine-rich repeat motifs—Clustering by physico-chemical properties

Screening the overall NLR dataset with LRRexpress yields a collection of ∼468,000 motifs in ∼31,100 sequences. As LRR domains may be found as standalone or part of many other protein classes only sequences containing regular NBS domains along with LRR domains larger than 9 repeats with no delineation ambiguities were retained for analysis, as described under methods.

The cleaned set of ∼61,700 LRR motifs from ∼6,850 seq were further clustered using unsupervised ML techniques in a space describing their hydropathy, charge and size, as described under methods. As various parts of the LRR horseshoe were shown to be involved in different types of internal and external interactions (Wang et al., 2019a; Ma et al., 2020) motifs were further grouped according to their position in the repeat ladder for 4,101 CNLs, 2,671 TNLs, and 84 RNLs.

**FIGURE 6:**
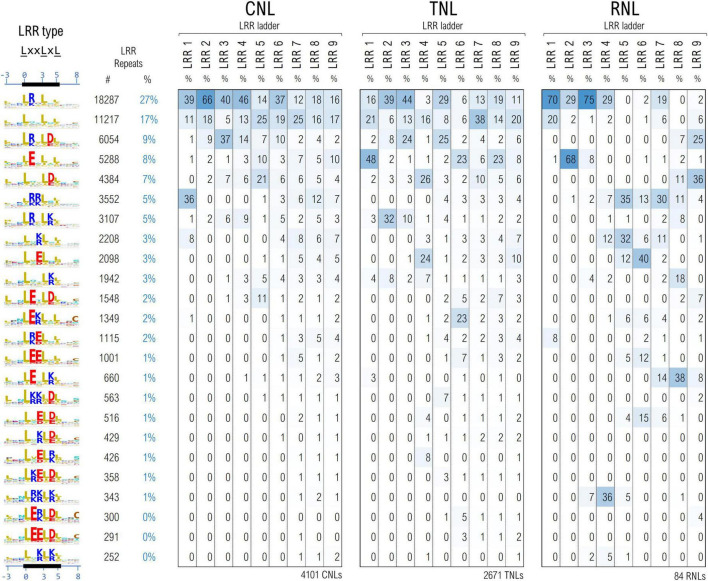
Clustering of the LRR motifs in a physico-chemical properties embedding—top 24 clusters and their distribution among position in the LRR domain ladder in the three NLR classes. Displayed values are percentages normalized vertically, with respect to the total number of repeats in the given position of the ladder. Amino acids are colored as described in Figure 3 caption.

Figure 6 shows the top 24 largest LRR motif clusters and their distribution in the LRR ladder for the three NLR classes. The most frequent pattern displays lysine and arginine in position 1 which is mainly found within the first 4–6 repeats of the analyzed NLRs. In general, the first 4–6 repeats of all three NLR classes show a high preference for positively charged LRR motifs, especially arginine. As can be seen from Figure 6, the cluster separation is mainly driven by the distribution of charge features in various solvent-exposed positions of the LxxLxL pattern.

In general more bulky charged amino acids, R&E, are preferred in positions 1 and 2 of the LxxLxL motif, while the lighter ones K&D are more frequent in position 4 of the motif. Remarkably Arginine in position 1 of the motif is dominant in the first four CNL repeats, in repeats 2 and 3 in TNLs and in repeats 1, 3 and 4 in RNLs. By contrast, aspartic acid in position 4 starts shaping up above repeat 3. Interestingly clusters with glutamic acid in position 1 are dominant in the first TNL and second RNL repeat in contrast with CNLs. Aside from the presence of Glu in the first TNL/RNL repeats, Figure 6 also indicates other interesting differences among the three NLR classes – for instance, while the second uncharged cluster shapes up quite frequently (>15%) and evenly in all CNL/TNL repeats, this is almost absent in RNLs with the exception of the first repeat (<4%).

The CNL clustering data are consistent with the experimental reports indicating that at least in some systems such as ZAR1, Rx, SW5 charge complementarity between the first 4-6 LRR repeats and the CC and NBS domains is involved in CC-LRR and NBS-LRR interfacing and has a critical role in correct CNL functioning (Slootweg et al., 2013; de Oliveira et al., 2018; Wang et al., 2019b).

#### Interdomain correlations in CNL receptors

**FIGURE 7:**
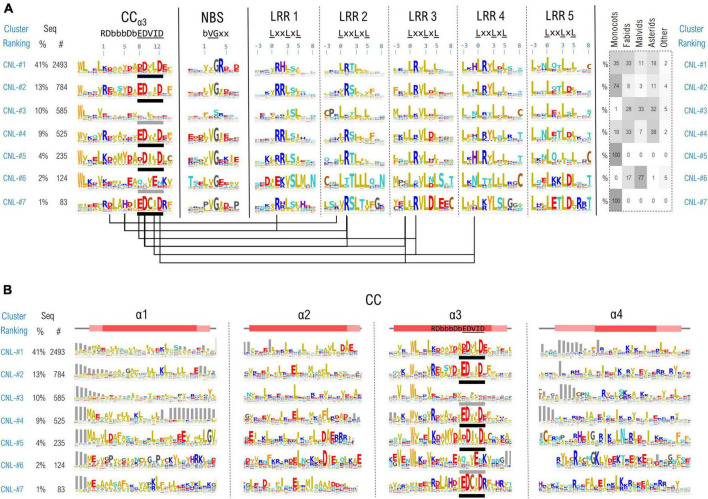
**(A)** CNL proteins clustering based on the regions confining the CC extended EDVID (α3 helix) and the first 5 LRR motifs. Motif variability is expressed as relative entropy displayed as letter height (the higher the more conserved). The taxonomic distributions of each cluster is depicted in the right panel. The ZAR1 CC-LRR contacts within 5Å are mapped below the motif consensus (PDB: 6j5w). **(B)** Zoom in on the conservation profile of the CC domain of the top CNL clusters from **(A)**.

Due to their overall fold or to the conformational changes taking place during NLR functioning, the individual domains might be brought close in space, permanently or transiently. Inter-domain contacts could leave traces in correlations between motifs which are the most invariant regions of this highly diverse class of proteins. To identify such correlations we simultaneously clustered all the 11 NLR motifs in the case of CNLs, mapping results onto the recently released structural data (Wang et al., 2019a,b). As CNLs represent the largest group of NLR proteins (∼14,100 CNLs vs. ∼5,100 TNLs and ∼400 RNLs in the analyzed set) and were shown to host a relatively conserved region readily detectable with ML techniques—the “extended EDVID” motif—we separated the main groups of this class of NLRs using clustering techniques described under the methods. The best cluster separation is reached at ∼37% identity where around 80% of the sequences do gather into the top seven CNL clusters. As can be seen from Figure 7A, five out of the seven main clusters: CNL#1, 2, 4, 5, and 7 display a strong charge complementarity between the EDVID and the first 5 LRR motifs, while clusters CNL#3 and 6 completely or partially lack strict EDVID pattern and charge match, with motifs being more diverse and neutral.

When mapping the motifs onto the ZAR1 cryo-EM structure the charge complementarity locates at the CC-LRR interface suggesting that sequences in CNL#1, 2, 4, 5, and 7 might adopt CC-LRR configurations similar to that seen in ZAR1. Some marked differences are noticeable though between clusters in LRR repeats 1–4 and the EDVID region which displays only two main conserved positions 9 and 12 in all these clusters.

Interestingly also the first two LRR motifs are quite irregular returning low probabilities by both LRRexpress and LRRpredictor, especially due to the unusual usage of amino acids in positions –3 (upstream) and L_0_ of the motif. This is related most likely to the involvement of these regions in CC-LRR interfacing which constitutes a stabilizing factor, while further repeats require a structural reinforcement provided by canonical leucines in these two positions.

The top largest CNL groups, clustered based on the most conserved 11 motifs (CC-EDVID, NBS, and LRR motifs) were next inspected with respect to the entire span of the CC domain (Figure 7B). Strong consensus signatures are observed solely within the third helix (α3)—with tryptophan upstream the extended EDVID motif highly conserved in most CNL groups. By contrast, the remaining three helical segments display a high sequence and length variability, preserving though the hydrophobic positions involved in coil-coiled zipping.

Among these, the first helix (α1) displays the highest variability which may be linked to both NLR localization and functioning. For instance, a general more hydrophobic profile is particularly seen in the CNL groups displaying canonical EDVID signatures. By contrast, groups lacking a strict EDVID pattern and CC-LRR charge match (CNL-#3&#6), display also a significantly higher sequence and length variability in α1.

The “MADA” motif in its extended form “MA(D/E)AxVSFxVxKLxxLLxxEx” (Adachi et al., 2019), also present in ZAR1 is seen mainly in CNL groups #1&#4 which is consistent with Adachi et al. (2019) observation that this motif is found in around one fifth of plant CNLs. While certain positions in this extended motif were experimentally shown to be essential in the activation and/or the protruding of the plasma membrane in ZAR1 (Baudin et al., 2019; Wang et al., 2019a; Bi et al., 2021) this is by no means an universal feature in CNLs. Other NLRs that display a N-ter α1 CC region highly non-homologous to ZAR1 attach to the membrane via N-myristoylation and/or palmitoylation such as RPS5 (Qi et al., 2012), while other NLRs such as Rx1 and RPS4, have a nucleo-cytoplasmic localization during their activation cycle (Tameling et al., 2012; Qi and Innes, 2013).

## Conclusion

Results presented herein indicate that NLRexpress—the bundle of 17 ML based predictors designed to identify CC, TIR, NBS and LRR specific sequence motifs—performs fast and accurately in identifying NLRs on large datasets and might be of use in overall genome, transcriptome or proteome screening. In addition, LRRexpress—the NLRexpress module for LRR motif prediction was tuned for general LRR detection and may be used as a fast and accurate standalone tool in identifying LRR domains and delineating repeats in other protein classes such as extracellular plant RLK/RLP or metazoan LRRs, making it a complementary tool to the existing DeepLRR (Liu et al., 2022), LRRpredictor (Martin et al., 2020a), LRRsearch (Bej et al., 2014), LRRfinder (Offord and Werling, 2013), etc.

NLRexpress was then use to detect motifs in a large set of ∼34,000 plant NLRs and cluster these proteins based on their motif properties using unsupervised methods in order to identify correlations within and between the canonical domains shaping up underneath the NLR significant diversity.

All in all, NLRexpress is designed as a flexible bundle for the fast prediction of main NLR domains in large sequence datasets. Using LRRexpress alone will pinpoint all proteins displaying LRR domains, most of which are immune system receptors. Using it in conjunction with NBSexpress will identify all NLRs irrespective to their group, while adding the CCexpress and/or TIRexpress modules will bring further information related to the main NLR groups—TNL and CNL.

## Data availability statement

The datasets and source code presented in this study can be found in online repositories. The names of the repository/repositories and accession number(s) can be found below: all training models and datasets can be found as tar.gz archives at https://nlrexpress.biochim.ro/documentation.php within the download section. The source code of NLRexpress can be found at: https://github.com/eliza-m/NLRexpress.

## Author contributions

A-JP and AG conceived the research framework. A-JP, AG, and LS supervised the work. EM carried out software implementation, validation, and data analysis. All authors contributed to analysis and interpretation of the data and writing of the manuscript, read, and approved the final manuscript.
