# Supplementary material for: NLRexpress—A bundle of machine learning motif predictors—Reveals motif stability underlying plant Nod-like receptors diversity
Source: Front Plant Sci. 2022 Sep 15;13:975888. doi: 10.3389/fpls.2022.975888 (PMC9519389; doi:10.3389/fpls.2022.975888)
Supplement: Supplementary file 1 [file Data_Sheet_1.PDF]

Supplementary Material

Supplementary Table 1

	Motif	Set	Out-of-sample							In-sample						
			TN	FP	FN	TP	Precision	Recall	Fscore	TN	FP	FN	TP	Precision	Recall	Fscore
CC	Extended EDVID (RDbbbDbEDbbD)	CV 1	13065	4	3	89	0.957	0.967	0.962	39126	1	0	274	0.996	1.000	0.998
		CV 2	12886	3	4	88	0.967	0.957	0.962	39304	3	0	274	0.989	1.000	0.995
		CV 3	12943	2	2	89	0.978	0.978	0.978	39249	2	0	275	0.993	1.000	0.996
		CV 4	13292	1	3	88	0.989	0.967	0.978	38902	1	11	264	0.996	0.960	0.978
		Test	12739	2	6	85	0.977	0.934	0.955	52194	2	1	365	0.995	0.997	0.996
TIR	βA (FbSFRGEDbR)	CV 1	17027	0	0	97	1.000	1.000	1.000	51117	0	0	292	1.000	1.000	1.000
		CV 2	16961	0	0	96	1.000	1.000	1.000	51183	0	1	292	1.000	0.997	0.998
		CV 3	16963	0	0	98	1.000	1.000	1.000	51181	0	0	291	1.000	1.000	1.000
		CV 4	17193	0	0	98	1.000	1.000	1.000	50951	0	0	291	1.000	1.000	1.000
		Test	17078	0	1	96	1.000	0.990	0.995	68144	0	0	389	1.000	1.000	1.000
	αA (xxFbSHL)	CV 1	17320	0	0	98	1.000	1.000	1.000	51997	0	0	294	1.000	1.000	1.000
		CV 2	17253	0	1	97	1.000	0.990	0.995	52064	0	0	294	1.000	1.000	1.000
		CV 3	17257	0	1	97	1.000	0.990	0.995	52059	1	0	294	0.997	1.000	0.998
		CV 4	17487	0	1	97	1.000	0.990	0.995	51830	0	3	291	1.000	0.990	0.995
		Test	17371	0	1	97	1.000	0.990	0.995	69316	1	0	392	0.998	1.000	0.999
	βC (SRbxbbbF)	CV 1	17222	0	1	97	1.000	0.990	0.995	51702	1	0	294	0.997	1.000	0.998
		CV 2	17155	0	0	98	1.000	1.000	1.000	51769	1	0	294	0.997	1.000	0.998
		CV 3	17159	0	0	98	1.000	1.000	1.000	51765	1	0	294	0.997	1.000	0.998
		CV 4	17388	1	0	98	0.990	1.000	0.995	51536	0	0	294	1.000	1.000	1.000
		Test	17273	0	0	98	1.000	1.000	1.000	68924	1	0	392	0.998	1.000	0.999
	αC (WCLxEL)	CV 1	17418	0	0	98	1.000	1.000	1.000	52290	1	0	294	0.997	1.000	0.998
		CV 2	17351	0	0	98	1.000	1.000	1.000	52357	1	0	294	0.997	1.000	0.998
		CV 3	17355	0	0	98	1.000	1.000	1.000	52353	1	0	294	0.997	1.000	0.998
		CV 4	17584	1	0	98	0.990	1.000	0.995	52124	0	0	294	1.000	1.000	1.000
		Test	17469	0	0	98	1.000	1.000	1.000	69708	1	0	392	0.998	1.000	0.999
	βD-αD1 (VbPbFYxbDPSDVRxQ)	CV 1	16438	0	1	97	1.000	0.990	0.995	49350	1	1	293	0.997	0.997	0.997
		CV 2	16371	0	0	98	1.000	1.000	1.000	49417	1	1	293	0.997	0.997	0.997
		CV 3	16374	1	1	97	0.990	0.990	0.990	49413	1	0	294	0.997	1.000	0.998
		CV 4	16604	1	0	98	0.990	1.000	0.995	49184	0	0	294	1.000	1.000	1.000
		Test	16489	0	0	98	1.000	1.000	1.000	65787	2	0	392	0.995	1.000	0.997
	αD3 (WRxALxxbAxbxG)	CV 1	16732	0	0	98	1.000	1.000	1.000	50236	1	0	290	0.997	1.000	0.998
		CV 2	16667	0	2	94	1.000	0.979	0.990	50301	1	0	292	0.997	1.000	0.998
		CV 3	16670	0	0	97	1.000	1.000	1.000	50298	1	0	291	0.997	1.000	0.998
		CV 4	16899	1	0	97	0.990	1.000	0.995	50069	0	0	291	1.000	1.000	1.000
		Test	16783	0	0	98	1.000	1.000	1.000	66968	1	1	387	0.997	0.997	0.997
NBS	VG (bbGRE)	CV 1	62682	5	10	163	0.970	0.942	0.956	186537	14	28	487	0.972	0.946	0.959
		CV 2	62323	0	20	152	1.000	0.884	0.938	186915	0	69	447	1.000	0.866	0.928
		CV 3	62025	2	18	153	0.987	0.895	0.939	187210	1	51	466	0.998	0.901	0.947
		CV 4	62198	3	13	159	0.982	0.924	0.952	187026	11	26	490	0.978	0.950	0.964
		Test	62294	1	20	152	0.994	0.884	0.935	249233	5	54	634	0.992	0.922	0.956

	Motif	Set	Out-of-sample							In-sample						
			TN	FP	FN	TP	Precision	Recall	Fscore	TN	FP	FN	TP	Precision	Recall	Fscore
NBS	P-loop (GbGGbGKTT)	CV 1	61995	0	0	173	1.000	1.000	1.000	184486	0	0	516	1.000	1.000	1.000
		CV 2	61635	0	0	172	1.000	1.000	1.000	184846	0	0	517	1.000	1.000	1.000
		CV 3	61338	0	0	172	1.000	1.000	1.000	185143	0	0	517	1.000	1.000	1.000
		CV 4	61513	0	0	172	1.000	1.000	1.000	184968	0	0	517	1.000	1.000	1.000
		Test	61607	0	0	172	1.000	1.000	1.000	246481	0	0	689	1.000	1.000	1.000
	RNBS-A (FDbRbWbxVS)	CV 1	61821	1	6	167	0.994	0.965	0.979	183970	0	22	494	1.000	0.957	0.978
		CV 2	61459	4	2	170	0.977	0.988	0.983	184327	2	5	512	0.996	0.990	0.993
		CV 3	61166	0	9	163	1.000	0.948	0.973	184625	1	10	507	0.998	0.981	0.989
		CV 4	61341	0	5	167	1.000	0.971	0.985	184448	3	12	505	0.994	0.977	0.985
		Test	61429	6	2	170	0.966	0.988	0.977	245772	20	0	689	0.972	1.000	0.986
	Walker-B (KRFbbbbDDbW)	CV 1	62167	1	0	173	0.994	1.000	0.997	184998	4	4	512	0.992	0.992	0.992
		CV 2	61805	2	2	170	0.988	0.988	0.988	185361	2	2	515	0.996	0.996	0.996
		CV 3	61509	1	1	171	0.994	0.994	0.994	185657	3	4	513	0.994	0.992	0.993
		CV 4	61684	1	1	171	0.994	0.994	0.994	185482	3	4	513	0.994	0.992	0.993
		Test	61774	5	5	167	0.971	0.971	0.971	247166	4	12	677	0.994	0.983	0.988
	RNBS-B (KbbbTTR)	CV 1	62339	2	0	173	0.989	1.000	0.994	185511	7	1	515	0.987	0.998	0.992
		CV 2	61977	2	2	170	0.988	0.988	0.988	185877	3	5	512	0.994	0.990	0.992
		CV 3	61680	2	3	169	0.988	0.983	0.985	186171	6	0	517	0.989	1.000	0.994
		CV 4	61857	0	6	166	1.000	0.965	0.982	186001	1	26	491	0.998	0.950	0.973
		Test	61949	2	3	169	0.988	0.983	0.985	247854	5	9	680	0.993	0.987	0.990
	RNBS-C (LxxxExWxLF)	CV 1	61822	0	2	171	1.000	0.988	0.994	183970	0	4	512	1.000	0.992	0.996
		CV 2	61463	0	4	168	1.000	0.977	0.988	184329	0	3	514	1.000	0.994	0.997
		CV 3	61166	0	0	172	1.000	1.000	1.000	184626	0	0	517	1.000	1.000	1.000
		CV 4	61341	0	0	172	1.000	1.000	1.000	184451	0	0	517	1.000	1.000	1.000
		Test	61435	0	1	171	1.000	0.994	0.997	245792	0	0	689	1.000	1.000	1.000
	GLPL (GLPLA)	CV 1	62687	0	0	173	1.000	1.000	1.000	186550	0	0	516	1.000	1.000	1.000
		CV 2	62323	0	0	172	1.000	1.000	1.000	186914	0	0	517	1.000	1.000	1.000
		CV 3	62026	0	0	172	1.000	1.000	1.000	187211	0	0	517	1.000	1.000	1.000
		CV 4	62201	0	0	172	1.000	1.000	1.000	187036	0	0	517	1.000	1.000	1.000
		Test	62295	0	0	172	1.000	1.000	1.000	249237	0	0	689	1.000	1.000	1.000
	RNBS-D (CFbYCxLFP)	CV 1	61995	0	0	173	1.000	1.000	1.000	184486	0	0	516	1.000	1.000	1.000
		CV 2	61635	0	0	172	1.000	1.000	1.000	184845	1	0	517	0.998	1.000	0.999
		CV 3	61337	1	0	172	0.994	1.000	0.997	185143	0	0	517	1.000	1.000	1.000
		CV 4	61513	0	1	171	1.000	0.994	0.997	184968	0	0	517	1.000	1.000	1.000
		Test	61607	0	1	171	1.000	0.994	0.997	246480	1	0	689	0.999	1.000	0.999
	MHD (bHD)	CV 1	63033	0	1	172	1.000	0.994	0.997	187582	0	4	512	1.000	0.992	0.996
		CV 2	62667	0	1	171	1.000	0.994	0.997	187948	0	3	514	1.000	0.994	0.997
		CV 3	62369	1	1	171	0.994	0.994	0.994	188245	0	3	514	1.000	0.994	0.997
		CV 4	62545	0	5	167	1.000	0.971	0.985	188070	0	12	505	1.000	0.977	0.988
		Test	62636	3	0	172	0.983	1.000	0.991	250612	3	2	687	0.996	0.997	0.996
LRR	LRR (LxxLxL)	CV 1	23495	1	75	347	0.997	0.822	0.901	68030	5	195	987	0.995	0.835	0.908
		CV 2	25119	27	43	425	0.940	0.908	0.924	66345	40	63	1073	0.964	0.945	0.954
		CV 3	21500	8	50	320	0.976	0.865	0.917	70010	13	139	1095	0.988	0.887	0.935
		CV 4	21362	19	34	310	0.942	0.901	0.921	70099	51	90	1170	0.958	0.929	0.943
		Test	21435	33	29	367	0.918	0.927	0.922	91443	88	98	1506	0.945	0.939	0.942

Supplementary Table 1. Detailed *in-sample* and *out-of-sample* performance analysis during cross-validation and the final test stage for each motif predictor.

Supplementary Figure 1

A LRR-express on LRR domains from PDB

Predictor	Dataset	Precision (NLC)	Recall (NLC)	F1-score (NLC)	Precision (L)	Recall (L)	F1-score (L)
LRRexpress	PDB set*	0.986	0.805	0.887	0.984	0.880	**0.929**
LRRexpress	LRRpredictor test set**	0.990	0.667	0.797	0.989	0.739	**0.846**
LRRpredictor		0.928	0.860	0.893	0.915	0.899	**0.907**

* 178 LRR structures at 90% identity seq

**150 LRR repeats surroundings at 50% identity seq

B LRR-express behaviour on LRR-like architectures

Non-LRR solenoidal architectures*	Total positions	LxxLxL patterns	False positive predicted	NLR-express LRR probability (%) 0-10	10-20	20-30	30-40	40-50	50-60	60-70	70-80	80-90	90-100
Pectate lyase	38579	2163	7	38500	44	15	8	5	2	4	0	1	0
Trimeric LpxA	15241	1028	1	15229	8	2	0	1	0	1	0	0	0
Armadillo	28907	2130	0	28875	24	6	2	0	0	0	0	0	0
Ankyrin	33369	2672	1	33318	35	10	4	1	1	0	0	0	0

* 50 sequences/set

** **L** - hydrophobic amino acids (A,C, V, L, I, M, F, W, Y) ; **X** - any residue

C NLR-express vs LRRpredictor on different LRR containing protein classes

Plant CNL 500 seq

LRRpredictor \ LRR-express	20 - 40	40 - 60	60 - 100
20 - 40	69	21	100
40 - 60	41	26	179
60 - 100	157	114	7273

96.3% (60 - 100 row); 96.4% (60 - 100 column)

Plant RLK 499 seq

LRRpredictor \ LRR-express	20 - 40	40 - 60	80 - 100
20 - 40	14	5	38
40 - 60	18	9	54
60 - 100	70	59	7203

98.2% (60 - 100 row); 98.7% (80 - 100 column)

Vertebrate NLR 500 seq

LRRpredictor \ LRR-express	20 - 40	40 - 60	80 - 100
20 - 40	12	5	3
40 - 60	9	0	5
60 - 100	249	145	2991

99.7% (60 - 100 row); 88.4% (80 - 100 column)

Plant TNL 500 seq

LRRpredictor \ LRR-express	20 - 40	40 - 60	60 - 100
20 - 40	39	36	103
40 - 60	36	25	93
60 - 100	110	95	7158

97.3% (60 - 100 row); 97.2% (60 - 100 column)

Plant RLP 500 seq

LRRpredictor \ LRR-express	20 - 40	40 - 60	80 - 100
20 - 40	16	10	40
40 - 60	20	6	48
60 - 100	113	107	10191

99.1% (60 - 100 row); 97.9% (80 - 100 column)

Vertebrate TLR 354 seq

LRRpredictor \ LRR-express	20 - 40	40 - 60	80 - 100
20 - 40	13	4	17
40 - 60	13	8	27
60 - 100	186	123	4681

99.1% (60 - 100 row); 93.8% (80 - 100 column)

Supplementary Figure 1. LRRexpress behavior on other protein datasets: (A) LRR domains with known 3D structure: a set of 178 PDBs trimmed at 90% identity and the test set of LRRpredictor which contains 150 highly divergent LRR motifs (trimmed at 50% identity on a 16 aa

range around the motif) - both sets are from LRRpredictor (Martin et al., 2020). Performance scores - precision, recall and F1-score - are computed when considering only the core LRR motifs (L) or when including also the more variable N/C marginal motifs (NLC). **(B) Other non-LRR architectures that contain LRR-like patterns:** pectate lyase, trimeric, armadillo and ankyrin. The sets are from (Martin et al., 2020) and contain 50 sequences from each protein class. **(C) Different immune related protein classes which contain LRR domains**: plant cytosolic NLRs: CNL and TNL, extracellular RLK and RLP receptors and vertebrate NLR and TLR, taken from (Martin et al., 2020). The agreement between LRRexpress and LRRpredictor are shown next to each matrix.

References

Martin, E. C., Sukarta, O. C. A., Spiridon, L., Grigore, L. G., Constantinescu, V., Tacutu, R., et al. (2020). LRRpredictor—A New LRR Motif Detection Method for Irregular Motifs of Plant NLR Proteins Using an Ensemble of Classifiers. *Genes (Basel).* 11, 286. doi: 10.3390/genes11030286.
